# Physical plasma-triggered ROS induces tumor cell death upon cleavage of HSP90 chaperone

**DOI:** 10.1038/s41598-019-38580-0

**Published:** 2019-03-11

**Authors:** Sander Bekeschus, Maxi Lippert, Kristina Diepold, Gabriela Chiosis, Thomas Seufferlein, Ninel Azoitei

**Affiliations:** 1 ZIK plasmatis, Leibniz Institute for Plasma Science and Technology (INP Greifswald), Felix-Hausdorff-Str. 2, 17489 Greifswald, Germany; 2 Center for Internal Medicine I, University Hospital of Ulm, Albert-Einstein-Allee 23, 89081 Ulm, Germany; 3 Department of Molecular Pharmacology and Chemistry, Memorial Sloan-Kettering Institute New York, New York, NY USA

## Abstract

HSP90 is a ubiquitously expressed molecular chaperone implicated in the correct folding and maturation of a plethora of proteins including protein kinases and transcription factors. While disruption of chaperone activity was associated with augmented cancer cell death and decreased tumor growth both *in vitro* and *in vivo*, the regulation of HSP90 is not clearly understood. Here we report that treatment of cancer cells with cold physical plasma, an emerging and less aggressive tumor therapy, resulted in ROS generation which subsequently triggered the cleavage of HSP90. Notably, cleavage of HSP90 was followed by the degradation of PKD2, a crucial regulator of tumor growth and angiogenesis. Pre-sensitization of cancer cells with subliminal doses of PU-H71, an HSP90 inhibitor currently under clinical evaluation, followed by treatment with cold-plasma, synergistically and negatively impacted on the viability of cancer cells. Taken together, cold-plasma can be used in conjunction with pharmacologic treatment in order to target the expression and activity of HSP90 and the downstream client proteins implicated in various cancer cell capabilities.

## Introduction

Despite continuous advance in medicine, cancer is still a major threat to human society. Accordingly, many new avenues have been explored in the last decades to battle this disease. One of them is the utilization of reactive oxygen species (ROS) as signaling and damaging agents^1^. Among others, heat shock proteins (HSPs) are major gatekeepers that regulate ROS-driven apoptosis^2^. As cancer cells often present deregulated apoptosis pathways, HSPs were identified as interesting targets for tumor therapy^3^. Exposure of cancer cells to ROS generated by ascorbate-driven menadione redox cycling resulted in a partial cleavage of heat shock protein 90 (HSP90) and formation of a 70 kDa fragment^4^. HSP90 is a ubiquitously expressed chaperone implicated in proper folding of various protein kinases, nuclear receptors and transcription factors^5–7^. By regulating these so-called HSP90 “client” proteins, the chaperone participates in maintaining cellular homeostasis^8,9^. We recently reported that pharmacological inhibition of HSP90 by PU-H71 was not only associated with augmented cell death and decreased cancer cell proliferation but also lead to destabilization and subsequent proteasomal degradation of protein kinase D2 (PKD2)^10^. The serine-threonine kinase PKD2 and its sister isoforms PKD1 and PKD3 belong to the calcium/calmodulin-dependent protein kinase superfamily^11^ and are activated by various stimuli including phorbol esters, ROS, receptor tyrosine kinases, and hypoxia^12–14^. PKD2 was shown to be implicated in cancer cell growth, migration, invasion, and angiogenesis^14–16^.

ROS are not only generated as metabolic byproducts but also in many physical therapies for medicine such as ionization radiation and photodynamic therapy^17^. A recently emerging technology for ROS-based therapy is cold physical plasma. Plasmas are generated by energizing gases leading to partial ionization (dissociation of highly energetic electrons) and often high temperatures. Technical advance led to generation of so-called cold physical plasmas. These multicomponent systems comprise of electrical fields, multitude light emission, ions and electrons, and reactive species. The latter are assumed to be the main driver in anticancer effects of plasma treatment. Exposure to plasma led to induction of cell growth arrest in colorectal cancer^18^ and apoptosis in glioblastoma, colon carcinoma, breast cancer, and melanoma^18–20^. Other reports showed that exposure of Jurkat cells to cold-plasma resulted in the activation of pro-proliferative signaling molecules such as ERK1/2 and MEK1/2^21^. Sensing *et al*., proposed that generation of reactive oxygen species (ROS) after cold-plasma treatment induces apoptosis^22^. Indeed, it has been shown that ROS are harmful to cells by inducing senescence, cell cycle arrest and apoptosis^23^. All these results urge thorough investigation of the intimate molecular mechanisms triggering various cellular events by cold-plasma.

The aim of this study was to investigate the molecular mechanism behind the cell death triggered by cold plasma treatment. Our work shows that cold-plasma seems to orchestrate molecular signals in epithelial tumors involving HSP90 chaperone and its client PKD2. Moreover, these data indicate that HSP90 inhibitors such as PU-H71, currently under clinical evaluation in patients, synergize at subliminal doses with cold plasma treatment toward impaired tumor cell viability.

## Material and Methods

### Cell lines

MDA-MB-231, SW480, MCF-7, PC3 and NCF3 cancer cell lines were cultured either in DMEM or RPMI1640 medium supplemented with 10% fetal bovine serum, 2% glutamine, and 1% penicillin/streptomycin (all Sigma, Germany). Cancer cell lines were obtained from DSMZ-German Collection of Microorganisms and culture cells (Braunschweig, Germany) or authenticated at the Multiplexion (Heidelberg, Germany). For subsequent experiments, cells were pre-incubated with either 50 nM of the pharmacological inhibitor of HSP90 8-[(6-iodo-1,3-benzodioxol-5-yl) sulfanyl]-9-[3-(propan-2-ylamino) propyl] purin-6-amine inhibitor PU-H71 or vehicle control for 4 h. PU-H71 was synthesized and characterized as previously reported^24^.

### Plasmids and lentiviral transduction

The pLenti6.2-V5-DEST-PKD2 over-expression vectors were generated using the pDONR-223-PKD2 entry clone from Addgene (PKD2, #23490). Knock-down of HSP90β was achieved using an HSP90-specific shRNA (#TRCN0000008748) from Open Biosystems. High-titer virus-containing supernatants of HEK 293FT cells after co-transfection of lentiviral vectors with pMD2.G and psPAX2 packaging plasmids were used for lentiviral-mediated transduction of cancer cells.

### Plasma treatment

**Figure 1:**
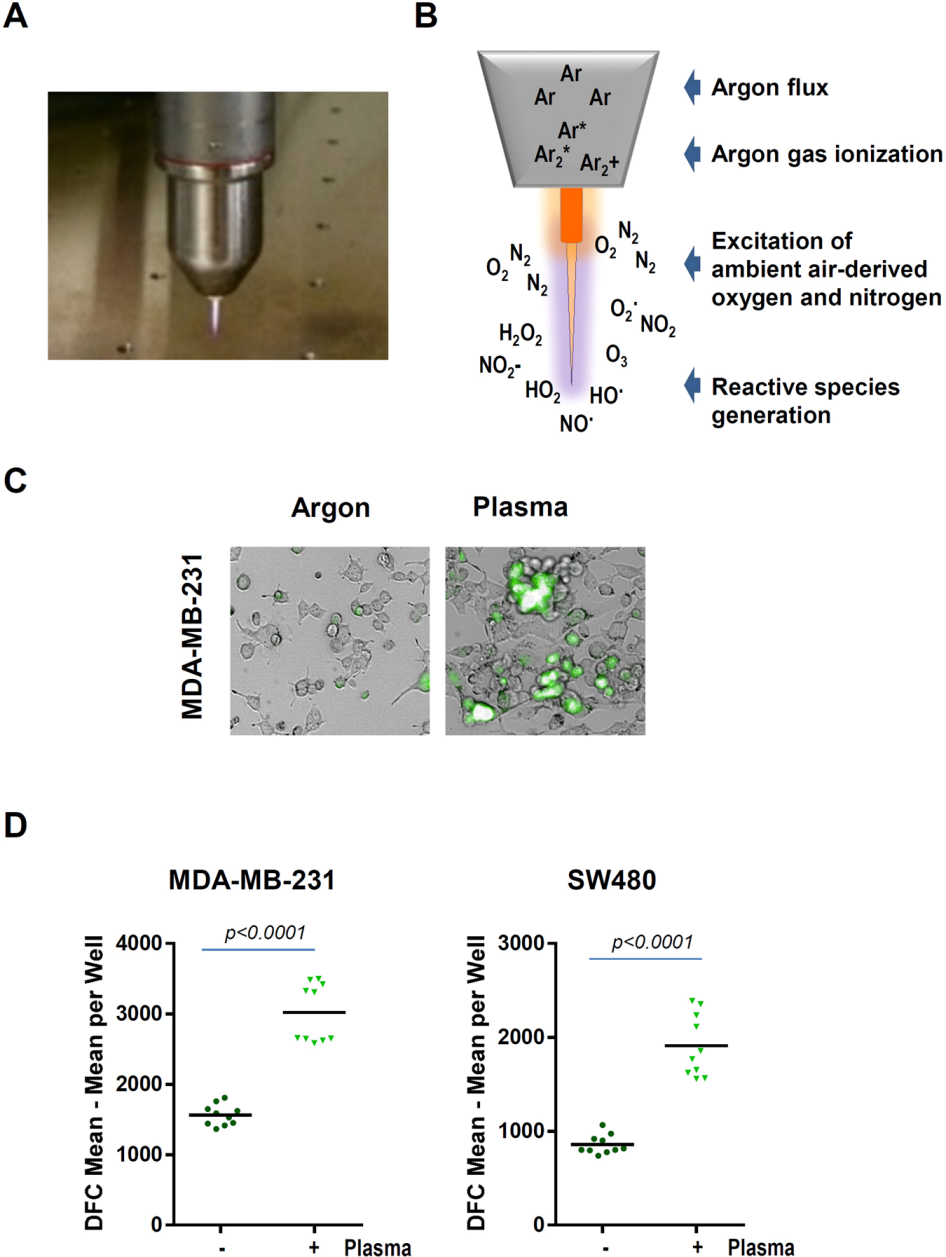
(**A**,**B**) The atmospheric pressure argon plasma jet kINPen and principle of reactive species generation. (**C**) Representative images of redox-sensitive dye CM-H_2_DCF-DA-loaded MDA-MB-231 cells in control and plasma-treated samples. (**D**) Quantitative image analysis of mean fluorescence intensity of the CM-H_2_DCF-DA redox-sensitive dye. Plasma treatment time was 60 seconds, image acquisition was performed 1 hour after plasma treatment.

For plasma treatment, 1 × 10^5^ cells were added per well in 24 well plates (Eppendorf, Germany) and allowed to adhere overnight. As plasma source, an atmospheric pressure argon plasma jet kINPen 11 (neoplas, Germany) was used (Fig. 1A). The kINPen 11 is technically similar to the kINPen MED that received approval as medical product for skin diseases. Argon gas (99.9999% pure; Air Liquide, France) was used to ignite the plasma that subsequently generated reactive species in the ambient air (Fig. 1B). The jet was hovered over the cells for the respective amount of time with a computer-programmed xyz-table (CNC, Germany).

### Detection of intracellular ROS

In the presence of ROS, the non-fluorescent CM-H_2_DCF-DA is converted to the green fluorescent form, when acetate groups are removed by intracellular esterases and intracellular oxidation occurs. Five micromolar of CM-H_2_DCF-DA (Thermo Fisher, USA) was added to the adherent cells. After 15 min of incubation, the extracellular dye was washed away. Cell cultures were subjected to plasma treatment. Bright field, digital phase contrast, and fluorescence were imaged using a high-content imaging device (Operetta CLS; PerkinElmer, Germany) with several fields of view per well using a 20x objective (Zeiss, Germany). Several thousand cells per condition were segmented upon signals in digital phase contrast images, and mean fluorescence intensity (MFI) per cell and well was analyzed using Harmony 4.6 software (PerkinElmer).

### Measurement of cell viability

Cell viability was assessed by incubating the cells in control and sample wells with 7-hydroxy-3H-phenox-azin-3-one-10-oxide (resazurin, final concentration 100 µM; Alfa Aesar, USA) for 4 h. In metabolically active cells, resazurin diffuses into the cells and is reduced to fluorescent resofurin. Fluorescence was quantified by using a microplate reader at λ_ex_ 535 nm and λ_em_ 590 nm (Tecan, Switzerland).

### Western blot analysis

Whole cell extracts were prepared using a lysis buffer containing 10 mM Tris-HCl, 5 mM EDTA, 50 mM NaCl, 50 mM NaF and 1% Triton X100 supplemented with Complete Protease inhibitor Cocktail (Roche). Lysates were subjected to SDS-PAGE and proteins transferred to PVDF membranes (Millipore, Massachusetts, USA). Membranes were blocked with 5% non-fat dry milk in phosphate buffered saline (PBS) containing 0.2% Tween 20 and incubated overnight at 4 °C with specific antibodies. For subsequent washes 0.2% Tween 20 in PBS was used. The following antibodies were used: HSP90β (D-19; Santa Cruz Biotechnology, #sc-1057), PKD2 (Bethyl Laboratories, #A300-073A), cleaved PARP (Cell Signaling, #9542 S), and β-actin (Sigma, #A1978).

### Tumor spheroids

3 × 10^3^ MDA-MB-231 cells were seeded in ultra-low-affinity round bottom plates (PerkinElmer), centrifuged, and grown for 72 h. Tumor spheroids were left untreated or exposed to plasma. After 24 h, 200 nM Sytox green (Life technologies, USA) was added and 15 z-stacks were imaged per well and spheroid. Using quantitative image analysis, spheroids stacks were merged into maximum intensity projections, segmented, and mean fluorescent intensity of Sytox green within spheroid area was assessed. Sytox green is a cell membrane-impermeable dye that enters only terminally dead cells to exhibit a strong fluorescence increase upon binding to DNA. Hence, it functions similar to e.g. propidium iodide (but with a different spectral properties) to stain dead cells.

### Statistical analysis

Statistical analysis as well as graphing was accomplished using Prism 7.03 (GraphPad Software, USA). Mean and statistical significance were calculated and analyzed using *t*-test. Levels of significances were indicated as follows: *p = 0.05, **p = 0.01, ***p = 0.001. p < 0.05 was considered significant. All data are representative of at least three experiments.

## Results

### Plasma-derived reactive species impair viability and augment cancer cell death

**Figure 2:**
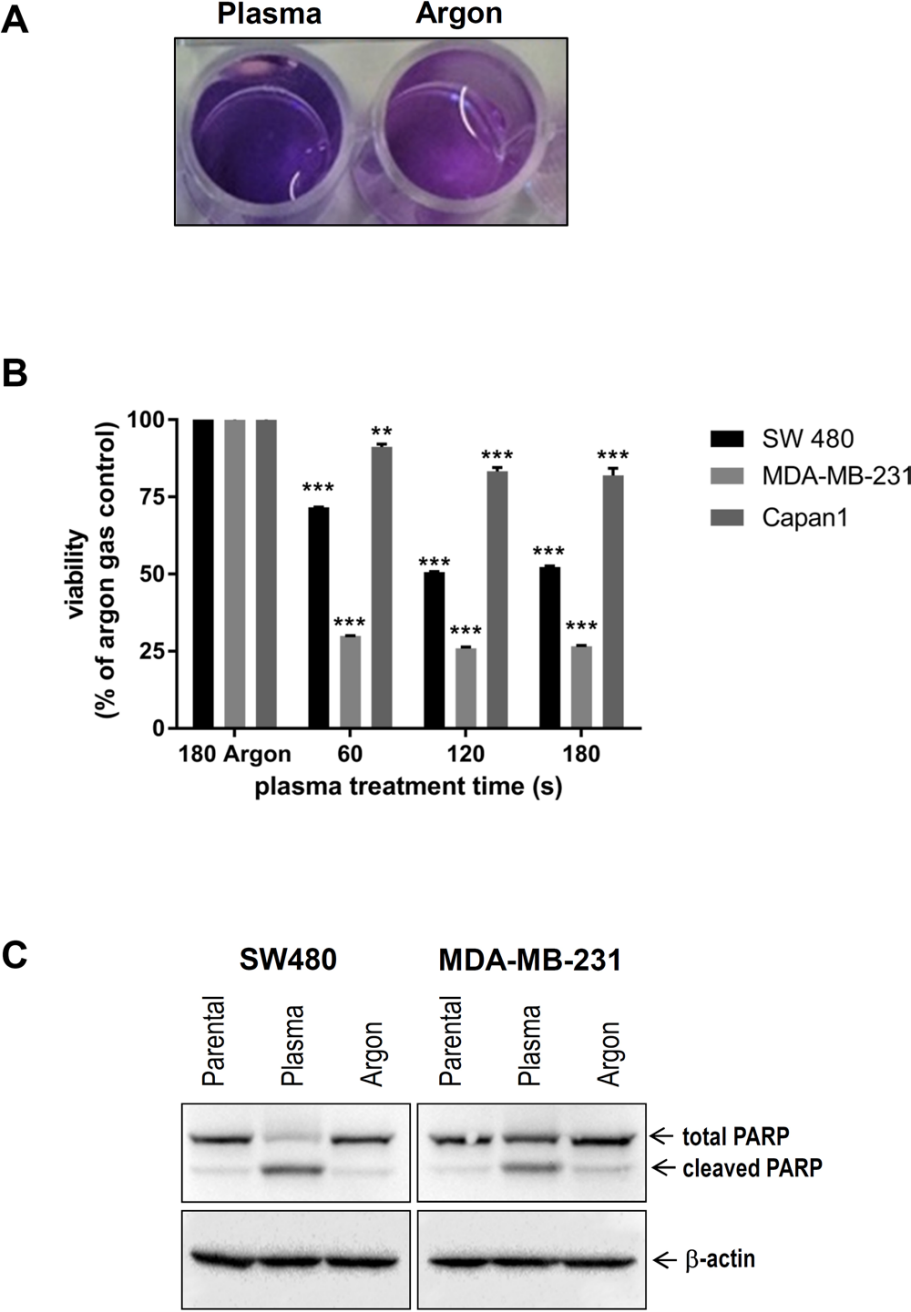
Cold plasma treatment results in impaired cancer cell viability and augmented apoptosis. (**A**) Representative image of resazurin assay, plasma treatment reduced transformation to pink resofurin indicated decrease in viability 24 hours after plasma treatment. (**B**) Viability of SW480, MDA-MB-231, and Capan1 in response to plasma treatment. MDA-MB-231 cells were most sensitive, followed by SW480, and Capan1, respectively. For each cell line, statistical coimparison (one.way analysis of variance with Dunnett post-test) of each plasma treatment time (60 s, 120 s, 180 s) compared to argon gas control (180 s) revealed significant (*p* < *0.01* or *p* < *0.001*) differences. (**C**) Twenty-four hours following argon gas or plasma treatment (both 60 s), lysates of MDA-MB-231 and SW480 were subjected to SDS-PAGE followed by incubation with PARP antibodies. β-actin was used as loading control.

Cold physical plasma is generated by an atmospheric pressure plasma jet (Fig. 1A). The plasma produces reactive species by ionizing a flow of argon gas that subsequently reacts with ambient air oxygen and nitrogen to form gas-phase reactive oxygen and nitrogen species (ROS/RNS), respectively (Fig. 1B). Cancer cells loaded with an intracellular fluorescent indicator of reactive species turnover were exposed to plasma, and cellular fluorescence was acquired (Fig. 1C). Quantification revealed significantly higher levels of intracellular ROS in plasma-treated cancer cells (Fig. 1D). Next, we investigated the effect of plasma intervention in the viability of various cancer cells. Resazurin assay demonstrated that treatment of cancer cells with plasma resulted in impaired survival as demonstrated by reduced transformation to pink resofurin (Fig. 2A). Furthermore, exposure of MDA-MB-231 breast and SW480 colon cancer cells to plasma jet-generated oxidative stress was associated with decreased viability (Fig. 2B). These results were substantiated in experiments with tumor spheroids (Suppl. Fig. 1). Capan1 pancreatic cancer cells showed resistance to plasma treatment, (Fig. 2B), very likely due elevated levels of SOD and decreased levels of CAT in these cells. In addition, western blot analysis with the apoptosis marker cleaved PARP, demonstrated that decreased cancer cell viability was paralleled by augmented cancer cell death (Fig. 2C).

### HSP90 is cleaved after treatment of cancer cells with cold plasma

**Figure 3:**
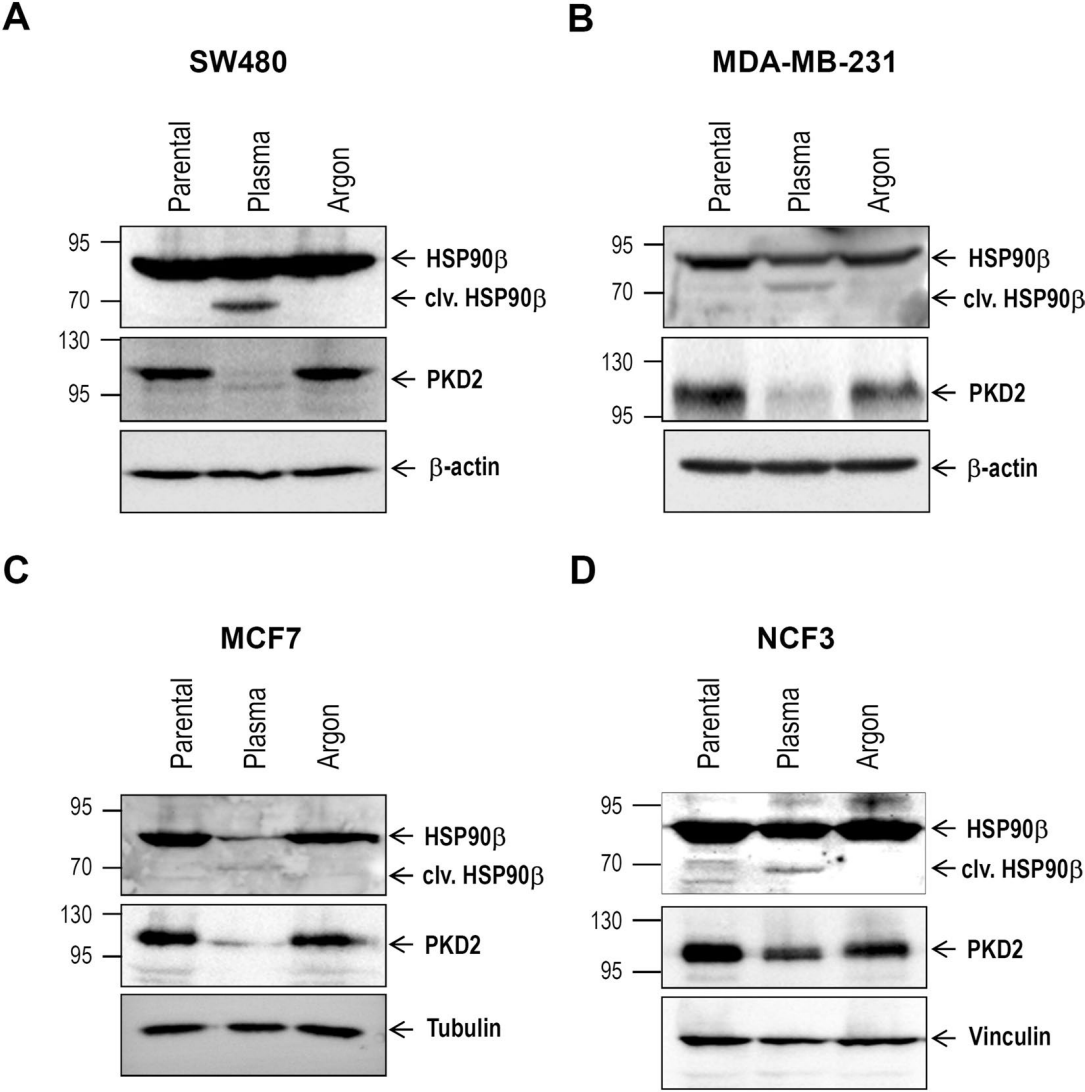
Cold plasma treatment is associated with cleavage of HSP90 chaperone and PKD2 degradation. (**A**–**D**) Various cancer cell lines were subjected to treatment with plasma jet (60 s). Twenty-four hours later, cleared lysates were used for western blot analysis in order to determine the HSP90 and PKD2 abundance.

HSP90, a cytoplasmic chaperone highly expressed in various cancers, has been reported to play an essential role in buffering stress conditions within the tumor microenvironment^25^. Beck and colleagues reported that exposure of cancer cells to ROS generated by ascorbate-driven menadione redox cycling resulted in cleavage of HSP90^4^. Interestingly, western blot analysis with lysates from various cancer cells treated with cold plasma, also revealed the same 70 kDa band (Fig. 3A–D), in line with the data of Beck and colleagues^4^. These results suggest that the chaperone is partially cleaved by the ROS generated upon plasma treatment which might be a potential tool in future cancer therapies. We previously reported that pharmacologic inhibition of HSP90 triggered proteasome-dependent degradation of PKD2^10^, a protein “client” involved in promoting cancer development. Interestingly, treatment of cancer cells with cold plasma not only resulted in cleavage of HSP90, but was also associated with PKD2 degradation (Fig. 3A–D). These results suggest that HSP90 cleavage-driven PKD2 degradation may contribute to the impaired proliferation and augmented cancer cell death observed after plasma treatment (Fig. 2A–C). Interestingly, cell death level following plasma treatment was comparable to that achieved upon lentiviral-mediated HSP90 knock-down (Suppl. Fig. 2A,B).

### Treatment with cold plasma boosts cell death in pharmacologically pre-sensitized cancer cells

**Figure 4:**
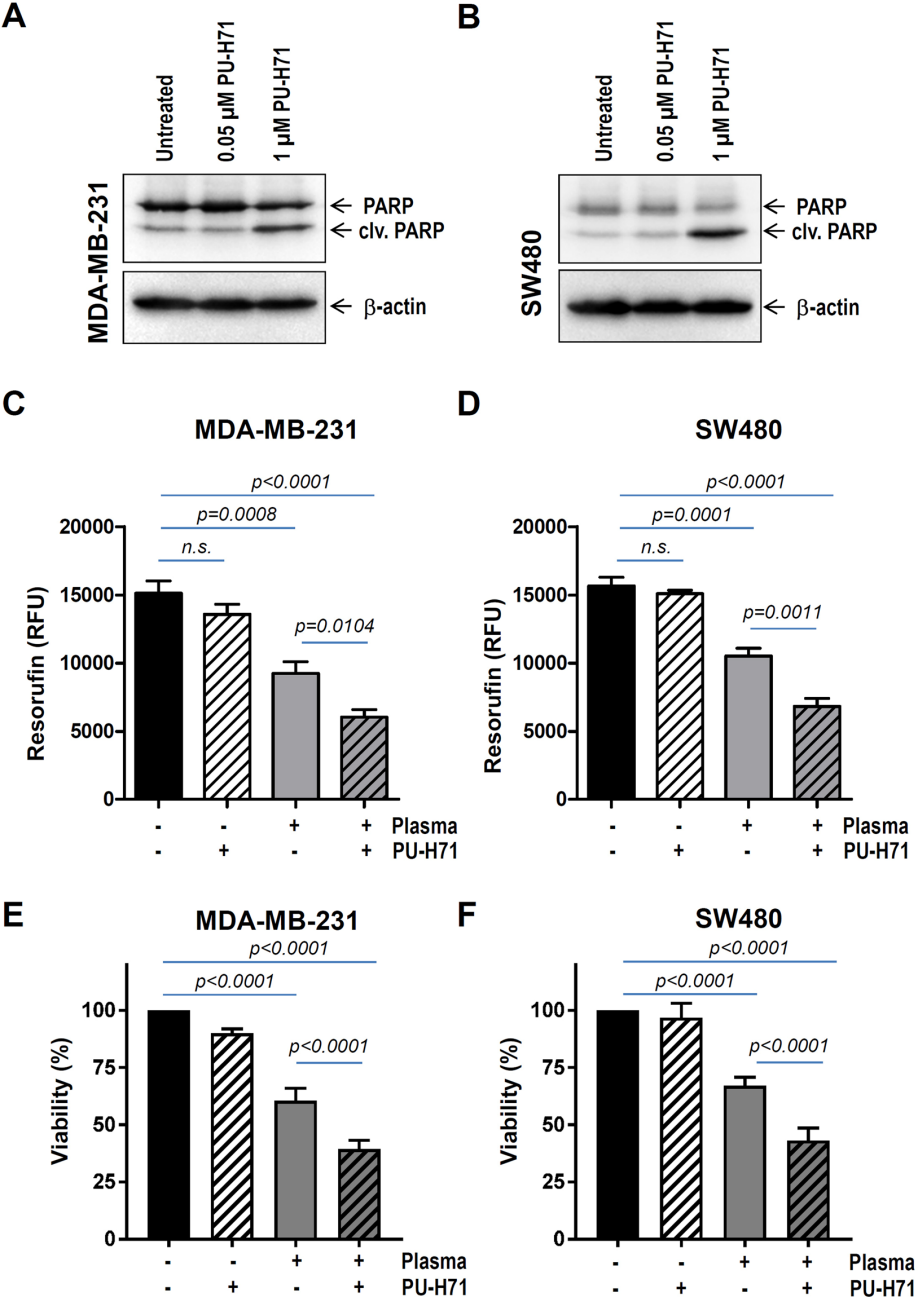
Cold plasma treatment boosts cell death in drug pre-sensitized cancer cells. (**A**) MDA-MB-231 breast and (**B**) SW480 colon cancer were treated for 24 hours with HSP90 inhibitor as indicated. Apoptosis was determined by examining the levels of cleaved PARP in western blot analysis. (**C**,**E**) MDA-MB-231 and (**D**,**F**) SW480 cancer cells were either incubated with 50 nM PU-H71 or subjected to plasma treatment (60 s) or both. A synergistic toxicity between PU-H71 and plasma treatment was observed at 24 h. RFU = relative fluorescence units.

Since HSP90 specifically maintains proper folding and function of various proteins mutated or over-expressed in human cancer^25–27^, several ATP-competitive HSP90 inhibitors are under clinical investigation^28–30^. Therefore, we sought to involve an HSP90 inhibitor, currently in the clinical trials, in a combined therapy with cold-plasma. Our previous studies showed that treatment of various cancer cells with 0.5 to 1 µM PU-H71 resulted in augmented cell death^10,31,32^. Here, we pre-incubated MDA-MB-231 breast and SW480 colon cancer cells with as less as 50 nM PU-H71 before plasma intervention. At this concentration, western blot analysis with cleaved PARP revealed no difference in cell death compared to untreated cells (Fig. 4A,B). On the other hand, 30 seconds cold-plasma treatment substantially reduced cancer cell viability (Fig. 4C–F). Most important, pre-sensitization of cancer cells with 50 nM PU-H71 was sufficient to significantly boost cell death triggered by cold-plasma therapy with up to around 60% (Fig. 4C–F). These results indicate a synergistic effect between the two therapies targeting the chaperone.

### Ectopic PKD2 is not sufficient to restore cancer cell viability after combined therapy

**Figure 5:**
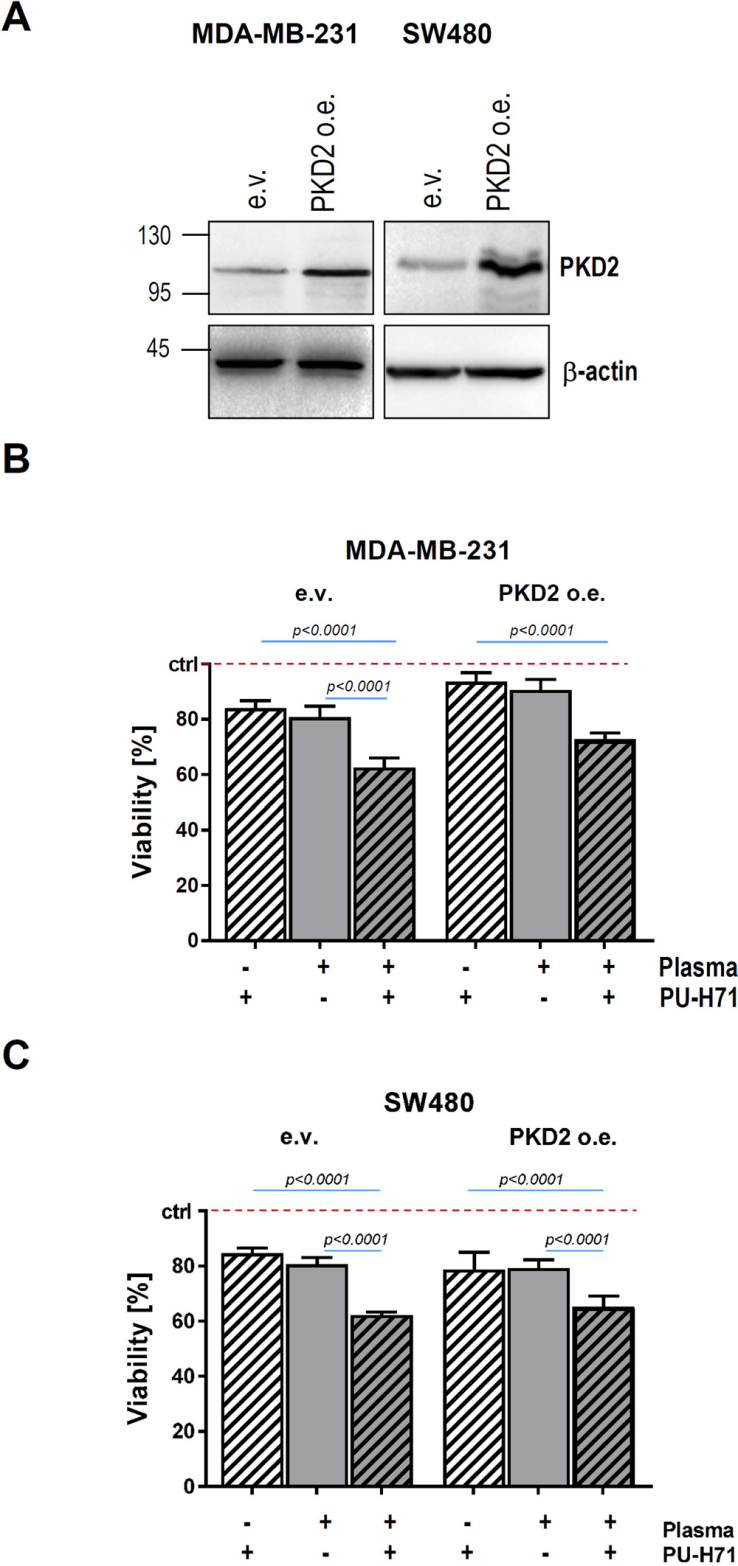
Ectopic PKD2 expression is not sufficient to restore cancer cell viability after treatment with cold plasma. Cancer cells were transduced with a PKD2 expression (PKD2 o.e.) or empty vector (e.v.). After selection with antibiotic, lysates of transduced cancer cells were prepared and SDS-PAGE was conducted with PKD2 antibody. β-actin was used as loading control. (**B,C**) MDA-MB-231 breast and SW480 colon cancer cells (e.v. as well as PKD2 o.e.) were treated with plasma (60 s) and/or 50 nM PU-H71, and cell viability was determined at 24 h.

Given that HSP90 inhibition results in decreased tumor growth in a PKD2-dependent manner^10^, we sought to investigate the magnitude of the kinase participation to cell death triggered through a combined intervention. Cancer cells transduced with an expression vector encoding PKD2 (PKD2 o.e.) or empty vector (PKD2 e.v.) (Fig. 5A) were subjected to pre-incubation with 50 nM PU-H71 for 24 hours before treatment with cold-plasma. Our results show that simple PKD2 over-expression is not enough to rescue cell viability after combined drug and plasma therapy (Fig. 5B,C). These results indicate that cell death following combined plasma and drug treatment is not a lone result of HSP90 cleavage and PKD2 degradation, respectively, as additional molecular mechanisms or molecules likely contribute to this cellular event as well. Investigation of another kinase reported to act as an HSP90 client, namely serine/threonine-kinase 33 (STK33)^31,32^, showed that cold-plasma treatment was also associated with STK33 degradation (Suppl. Figs 2B and 3A). The fact that PKD2 and STK33 represent just two of around 200 client proteins described until now for the chaperone, may very well reason the lack of restored viability after ectopic PKD2 expression alone.

## Discussion

Most existing drugs designed to target and kill cancer cells are harmful and poisonous to the human body as they also attack non-malignant cells, thus triggering serious side effects. Therefore, scientists are set to tailor and combine new therapies, less aggressive and invasive. In the last decade, several reports successfully documented the implication of cold-plasma in treatment of various tumors. While the results are promising, the intimate molecular mechanism of how cold-plasma therapy impacts on tumor cell viability remains elusive to date.

In this study we have demonstrated that treatment of cancer cells with cold-plasma results in generation of ROS, which negatively impacts on tumor cell proliferation and viability. Furthermore, plasma-driven ROS triggered the cleavage of HSP90 chaperone and subsequent degradation of its client PKD2, previously shown to be essential for cancer cell proliferation and viability. Finally, our study revealed that combined therapy using sub-minimal doses of HSP90 inhibitor and cold-plasma treatment was associated with decreased viability of cancer cells.

Cold physical plasmas are multicomponent systems with potential biological impact from e.g. UV and mild thermal radiation, electrons and ions, electric fields, and ROS/RNS. However, for effects in *in vitro* cultures, only few can be held accountable for the results observed. The UV-C portion of plasma penetrates liquids only 100 nm in depths^33^, thus cells underneath cell culture liquid are protected. The distance plasma-derived solvated electrons can travel in liquids is even lower and in the range of 2 nm^34^. Although the presence of positive ions in the gas phase of the plasma effluent has been directly shown^35^, measurements of solvated ions into the liquid (and their subsequent biological effects) is technically challenging. For thermal radiation, the jet was measured to have 37 °C at the tip of the effluent, making non-physiological heating of cells during plasma treatment unlikely. When the jet is operated in ambient air surrounding the plasma, electric fields contribute to its propagation to a minimal extent only^36^. Hence, while all of the above plasma parameters in principle are capable of having an impact on cells, their role in our setup is negligible. By contrast, ROS/RNS were shown to be the prime contributor in plasma-treated cells *in vitro*^37–39^.

**Figure 6:**
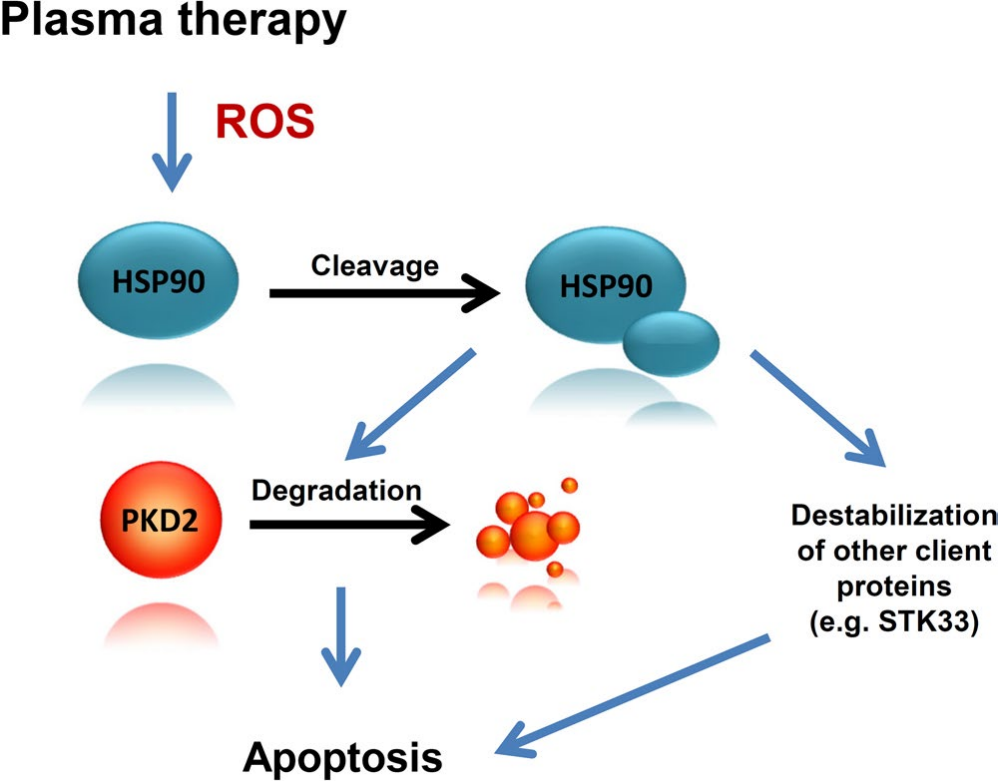
Cleavage of HSP90 and degradation of PKD2 following cold plasma treatment is associated with cancer cell death. Physical plasma treatment- generated ROS is followed by HSP90 cleavage and subsequent destabilization and degradation of PKD2. While PKD2 degradation plays an important role in cancer cell death, additional essential molecules such as STK33, also contribute to the apoptotic event. Furthermore, pre-treatment of cancer cells with subliminal doses of HSP90 inhibitor followed by cold plasma treatment boosts cell death in human cancer.

HSP90 orchestrates the stabilization and activation of around 200 protein clients, many of which are oncoproteins required for the acquisition and maintenance of the major cancer hallmarks. This renders HSP90 to be a crucial target when developing new strategies and drugs to inhibit chaperone’s activity and induce cancer cell death through the destabilization of client proteins. Beck and colleagues were able to provoke the oxidative cleavage of HSP90 upon generation of ROS by a Fenton-type reaction in proximity to the N-terminal nucleotide binding site of the chaperone^4^. The cleavage is biologically relevant as it was followed by K562 cell death^4^. The fact that cold-plasma generates ROS, for instance superoxide, peroxide and hydroxyl radicals^40^ prompted us to investigate, whether treatment of cancer cells with cold-plasma results in the cleavage of chaperone. To our best knowledge, this work is the first to show that cold-plasma treatment is associated with HSP90 cleavage. *In vitro* treatment of colon, prostate and breast cancer cells with cold-plasma resulted in 70 kDa fragment, in line with the previous data^4^. The next question was whether cleavage of HSP90 at the crucial site in the N-terminus responsible for chaperone’s activity, was associated with client degradation. Indeed, treatment with cold-plasma was associated with the degradation of PKD2, a protein shown in our laboratory to act as a HSP90 client^10^. These results suggest that one mechanism, by which cell death is promoted after plasma treatment, is represented by ROS-induced HSP90 cleavage and subsequent PKD2 degradation (Fig. 6). Intriguingly enough, cell death triggered by plasma-induced HSP90 cleavage-induced PKD2 destabilization was not restored by overexpressing PKD2. This suggests that additional chaperone client proteins might be involved in this process. Our investigations show that at least one additional client of HSP90, namely STK33, is involved in this scenario as plasma treatment also triggered its degradation (Fig. 6). The very likely involvement of many other client proteins in the cell death following HSP90 cleavage by plasma, reasons the lack of viability rescue in our experimental setup after attempting to overexpress PKD2 only. To note, cleavage of HSP90/degradation of PKD2 is only one within several molecular events following delivery of cold-plasma to cancer cells. Many of these death-triggering molecular events are not known or are barely understood.

Our recent results show that as less as 1 µM PU-H71 is sufficient to promote cell death as a result of HSP90 inhibition-triggered client degradation^10,31,32^. In an attempt to mimic sub-liminal drug doses in clinical setup we used for further experiments 50 nM PU-H71. At this concentration no cell death was detected upon cleaved PARP analysis. However, 50 nM was sufficient to sensitize cancer cells to plasma therapy, so that a synergistic effect between drug and plasma was achieved. This finding favours targeting HSP90 in a combinatorial therapy.

However, future studies using more tumor types and *in vivo* animal models are needed to provide information about the generalization of our finding and its relevance in biological systems.

## Supplementary information

Physical plasma-triggered ROS induces tumor cell death upon cleavage of HSP90 chaperone
