# Supplementary material for: Physical plasma-triggered ROS induces tumor cell death upon cleavage of HSP90 chaperone
Source: Sci Rep. 2019 Mar 11;9:4112. doi: 10.1038/s41598-019-38580-0 (PMC6412052; doi:10.1038/s41598-019-38580-0)

Supplementary Information

Physical plasma-triggered ROS induces tumor cell death upon cleavage of HSP90 chaperone

*Sander Bekeschus[1], Maxi Lippert[1#], Kristina Diepold[2#], Gabriela Chiosis[3], Thomas Seufferlein[2], *Ninel Azoitei[2],

[1] ZIK *plasmatis*, Leibniz Institute for Plasma Science and Technology (INP Greifswald), Felix-Hausdorff-Str. 2, 17489 Greifswald, Germany

[2] Center for Internal Medicine I, University Hospital of Ulm, Albert-Einstein-Allee 23, 89081 Ulm, Germany

[3] Department of Molecular Pharmacology and Chemistry, Memorial Sloan-Kettering Institute New York, NY, USA

***Corresponding authors:**

Ninel Azoitei: ninel.azoitei@uni-ulm.de (+ 49 731 500 45726)

Sander Bekeschus: sander.bekeschus@inp-greifswald.de (+49 3834 554 3948)

[#]These authors contributed equally

Suppl. Figure 1. Plasma treatment is toxic in MDA-MB-231 3D tumor spheroids. Control or plasma-treated (30s) spheroids were incubated at 24h with cell death dye Sytox green, (A) representative images, (B) quantitative image analysis of mean fluorescent intensity (MFI) within spheroid area.

Suppl. Figure 2. Lentiviral-mediated HSP90 knock-down in SW480 (A) and MDA-MB-231 (B) cancer cells was conducted. Lysates of cancer cells with abrogated HSP90 or subjected to plasma treatment (30s) were used for western blot analysis with cleaved PARP. β-actin was used as loading control.

Suppl. Figure 3. SW480 (A) and MDA-MB-231 (B) cancer cell lines were subjected to treatment with plasma jet (60s). Twenty-four hours later, cleared lysates were used for western blot analysis in order to determine the HSP90 and STK33 abundance. β-actin was used as loading control.

Suppl. Figure 4. SW480 cancer cells were transduced with PKD2 expression (PKD2 o.e.) or empty vector (e.v.), selected with the appropriated antibiotic before being subjected to plasma treatment (30s). Lysates of transduced cancer cells were prepared and SDS-PAGE was conducted with PKD2 antibody. β-actin was used as loading control.

Suppl. Figure 1

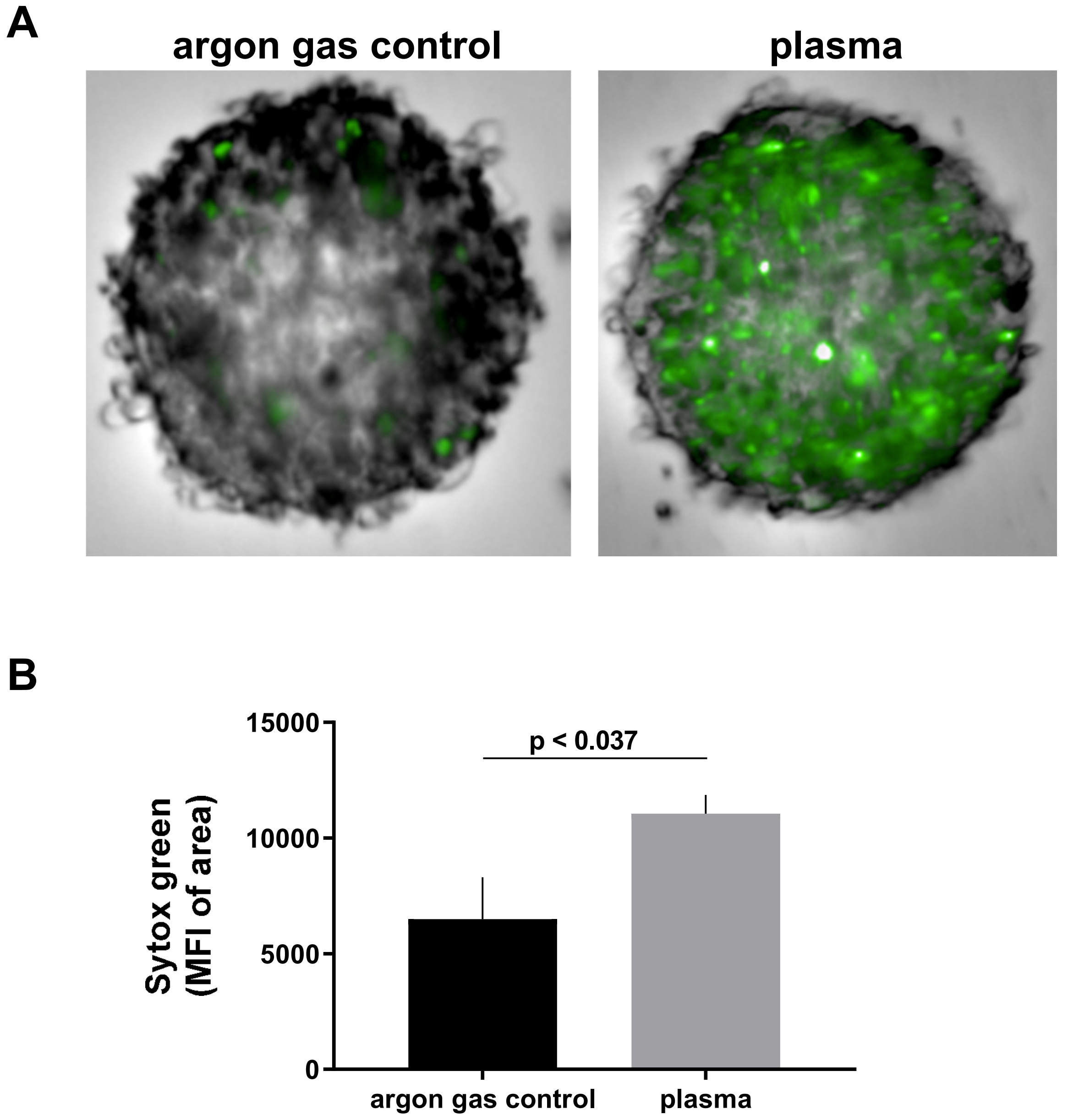

Suppl. Figure 2

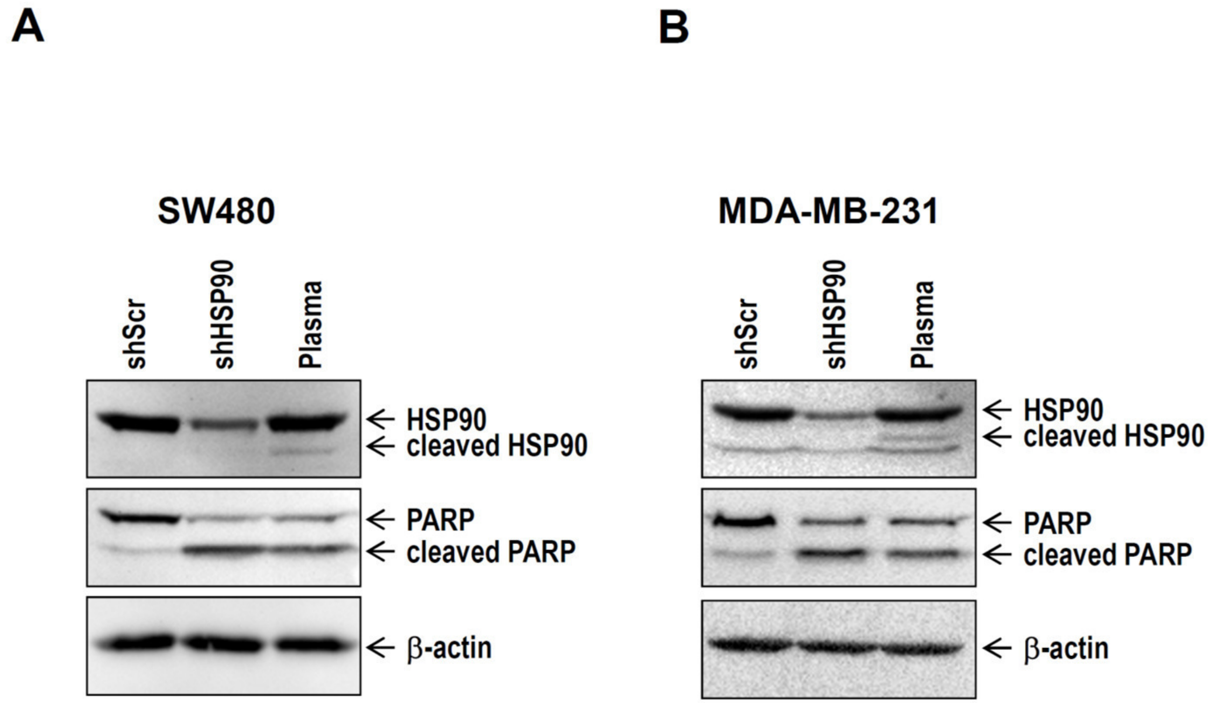

Suppl. Figure 3

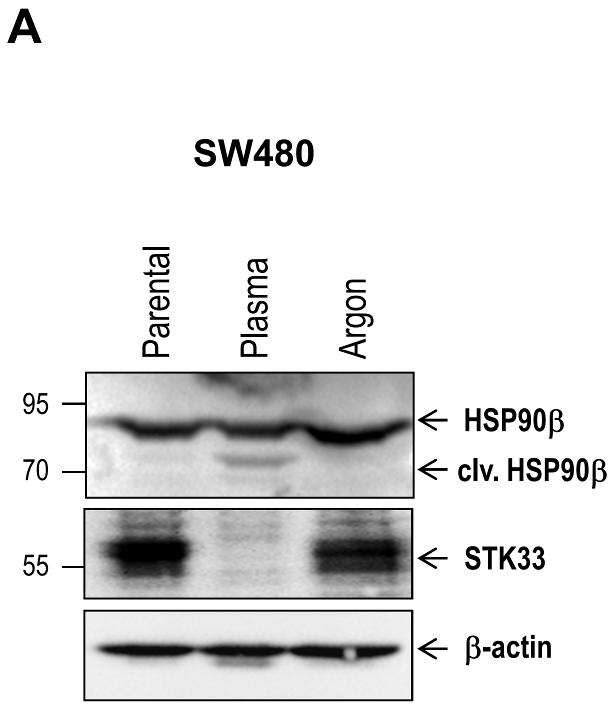

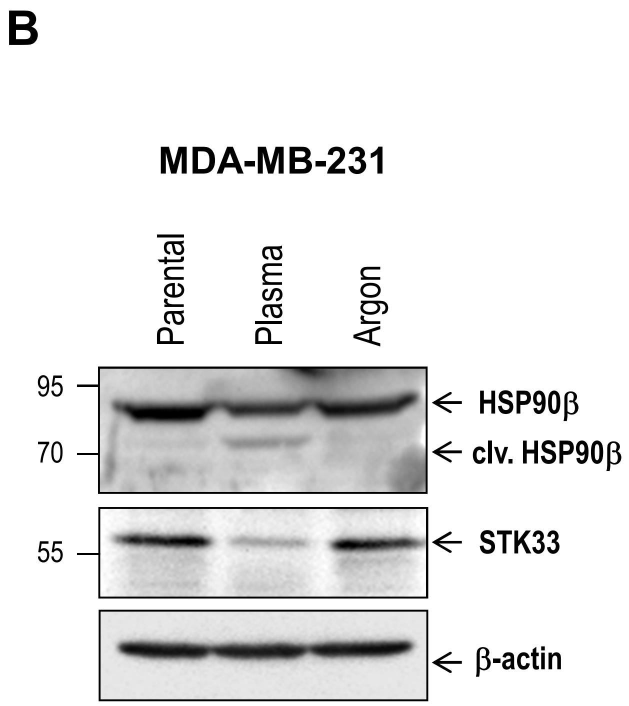

Suppl. Figure 4

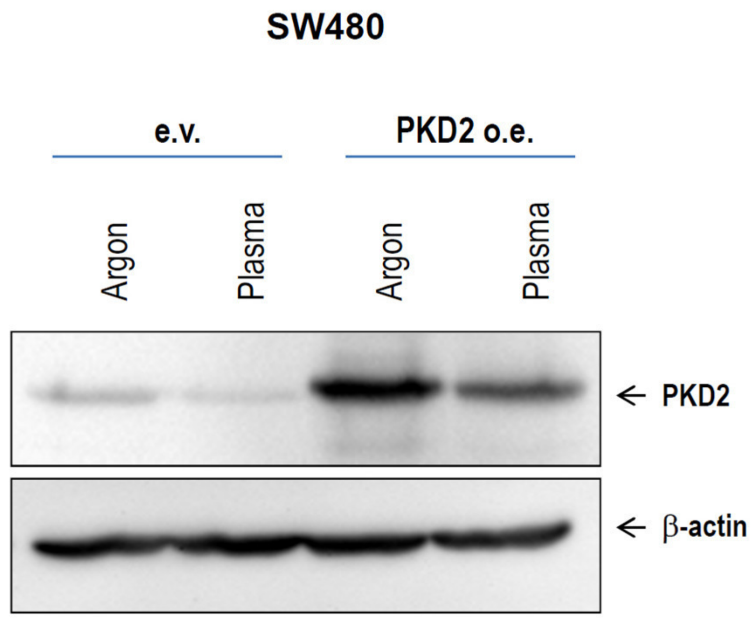

Figure 2

Full blots to

C

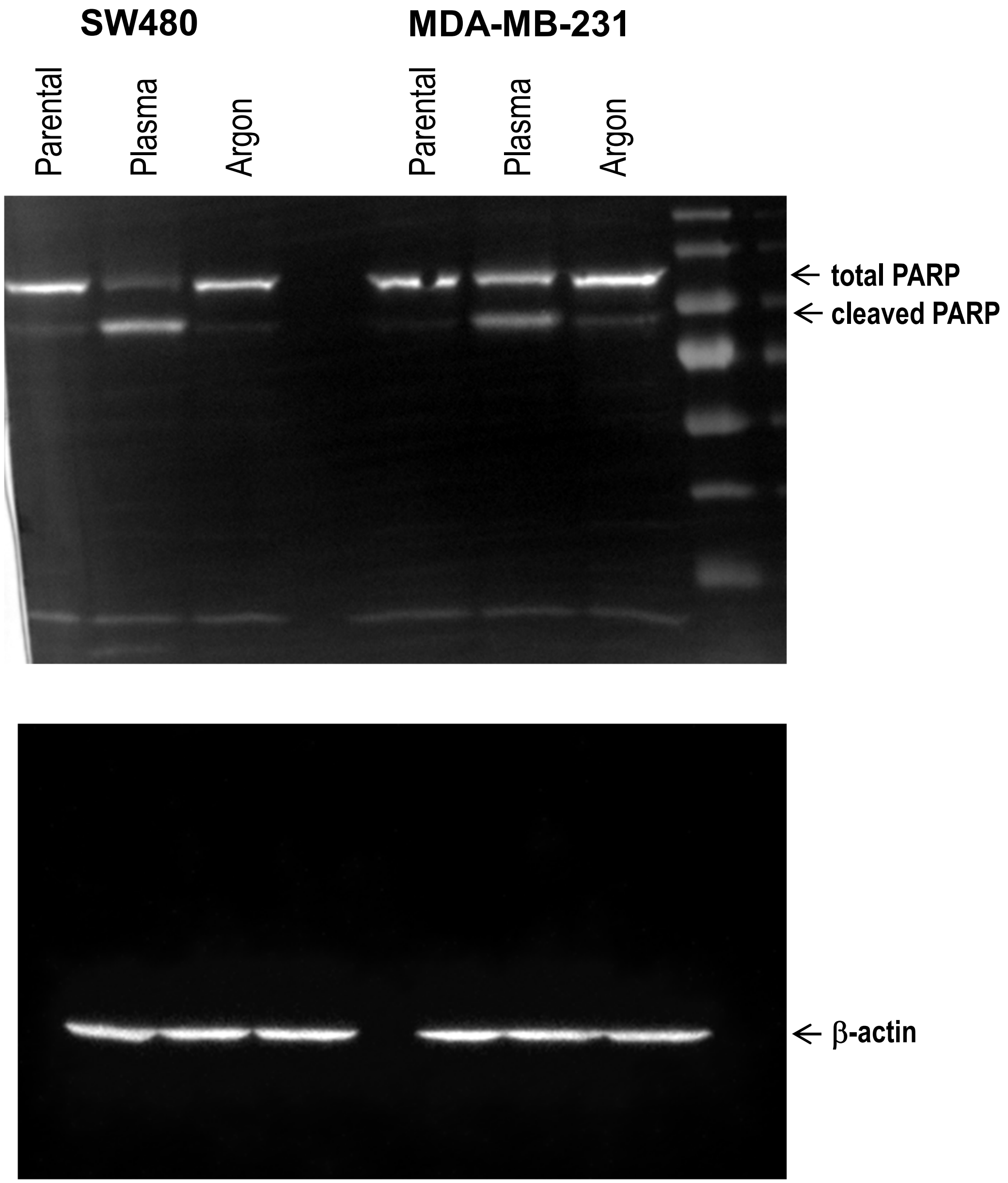

Figure 3

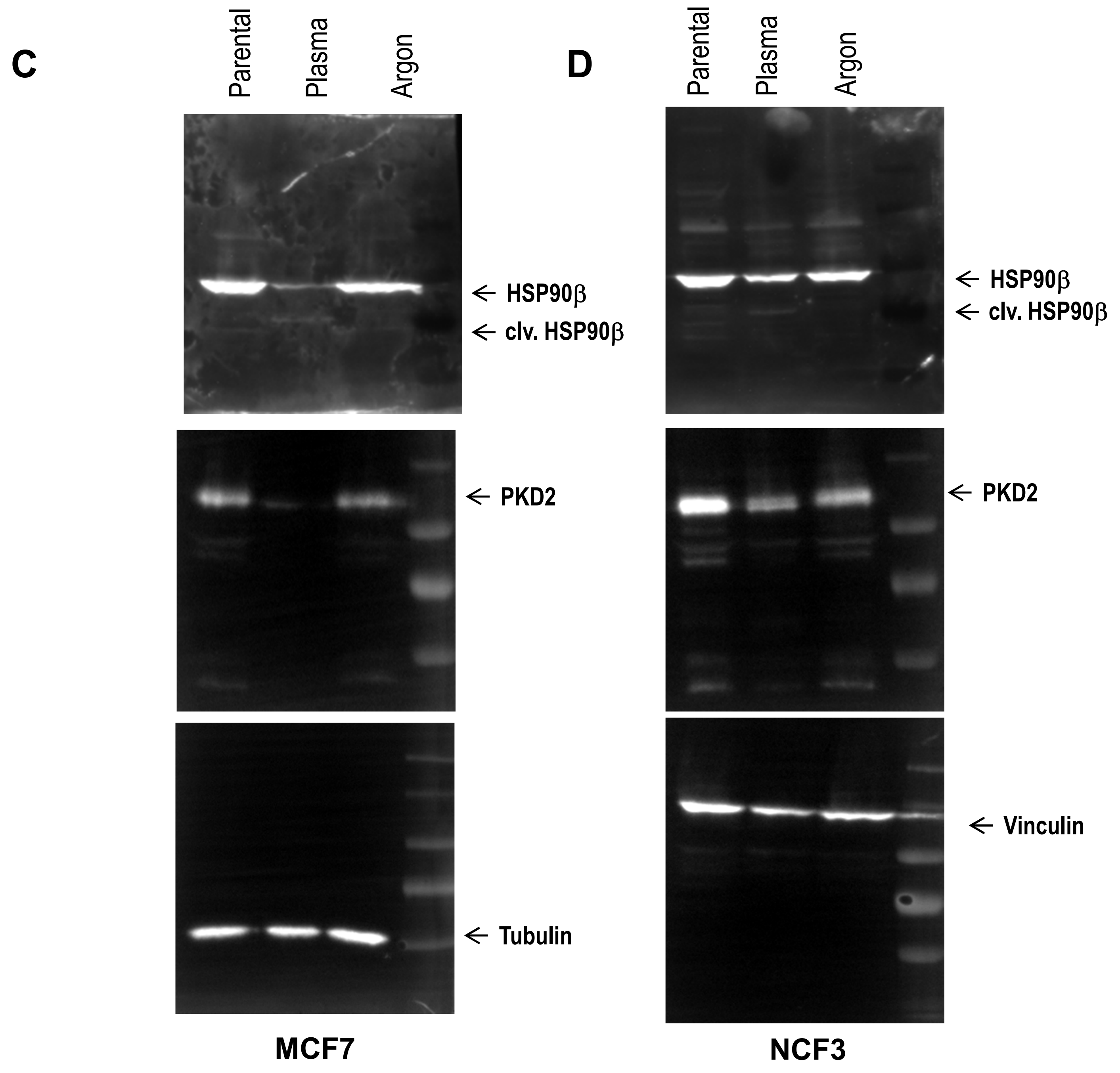

Full blots to

Figure 3

Full blots to

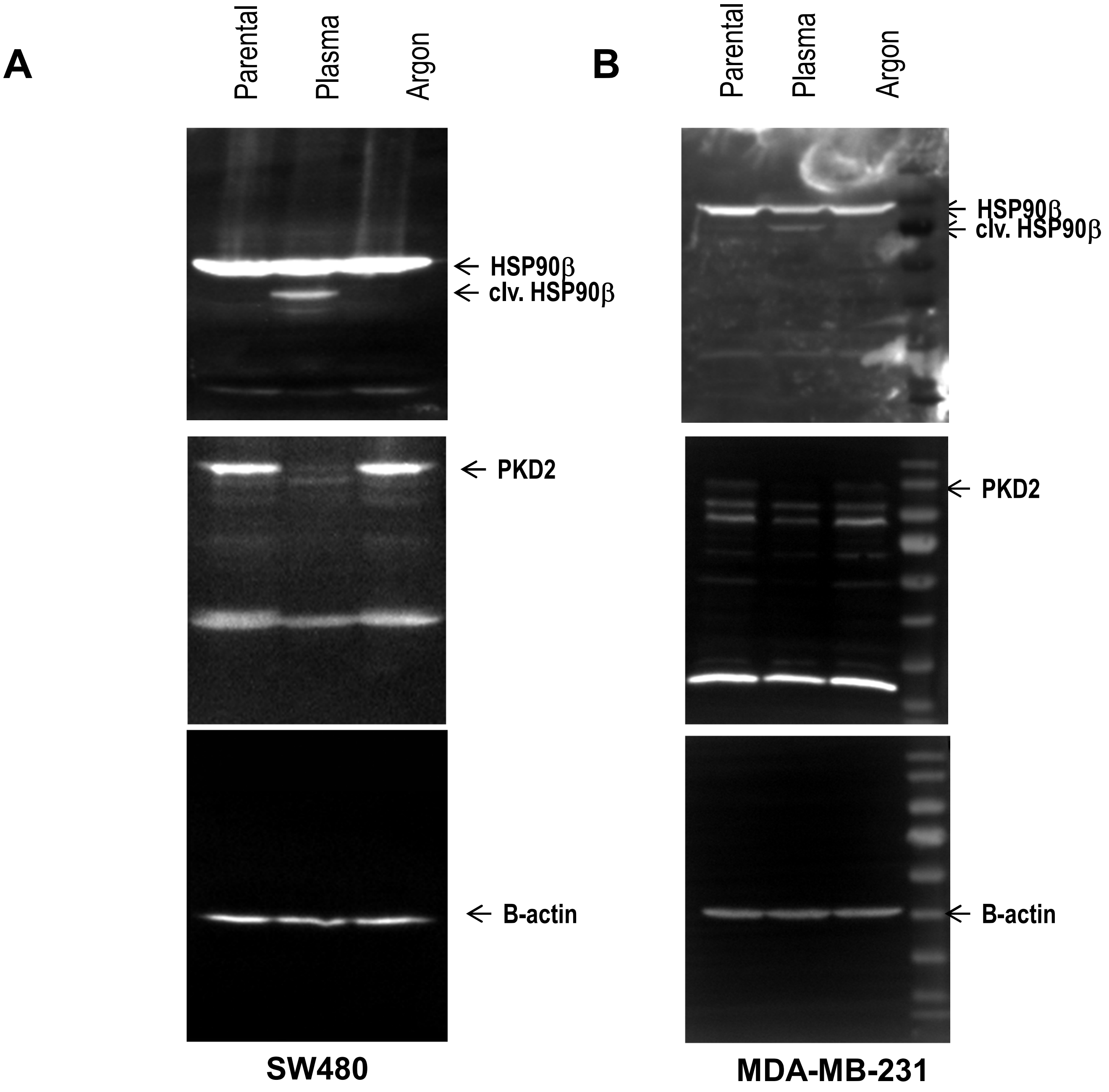

Figure 4

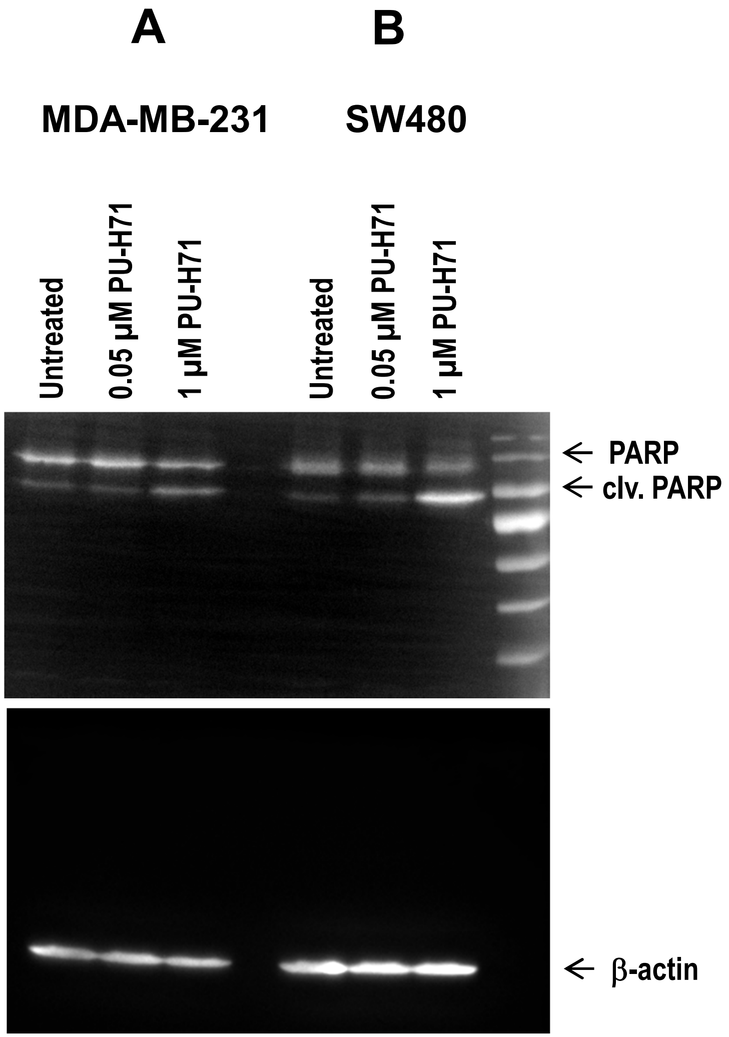

Full blots to

Figure 5

Full blots to

A

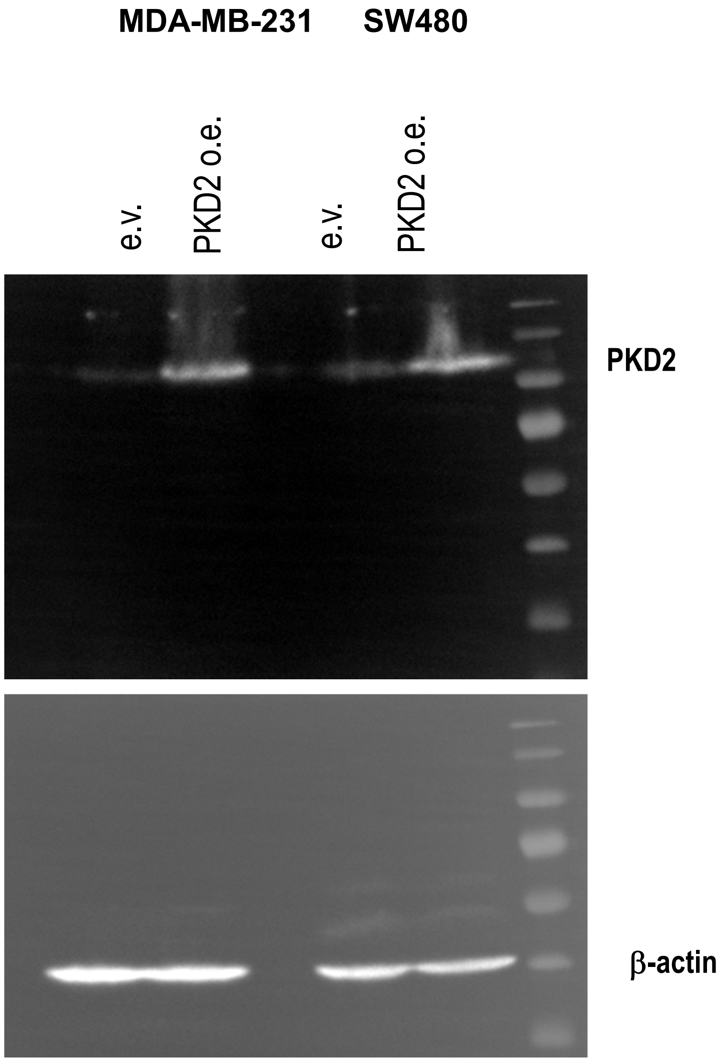

Suppl. Figure 2

Full blots to

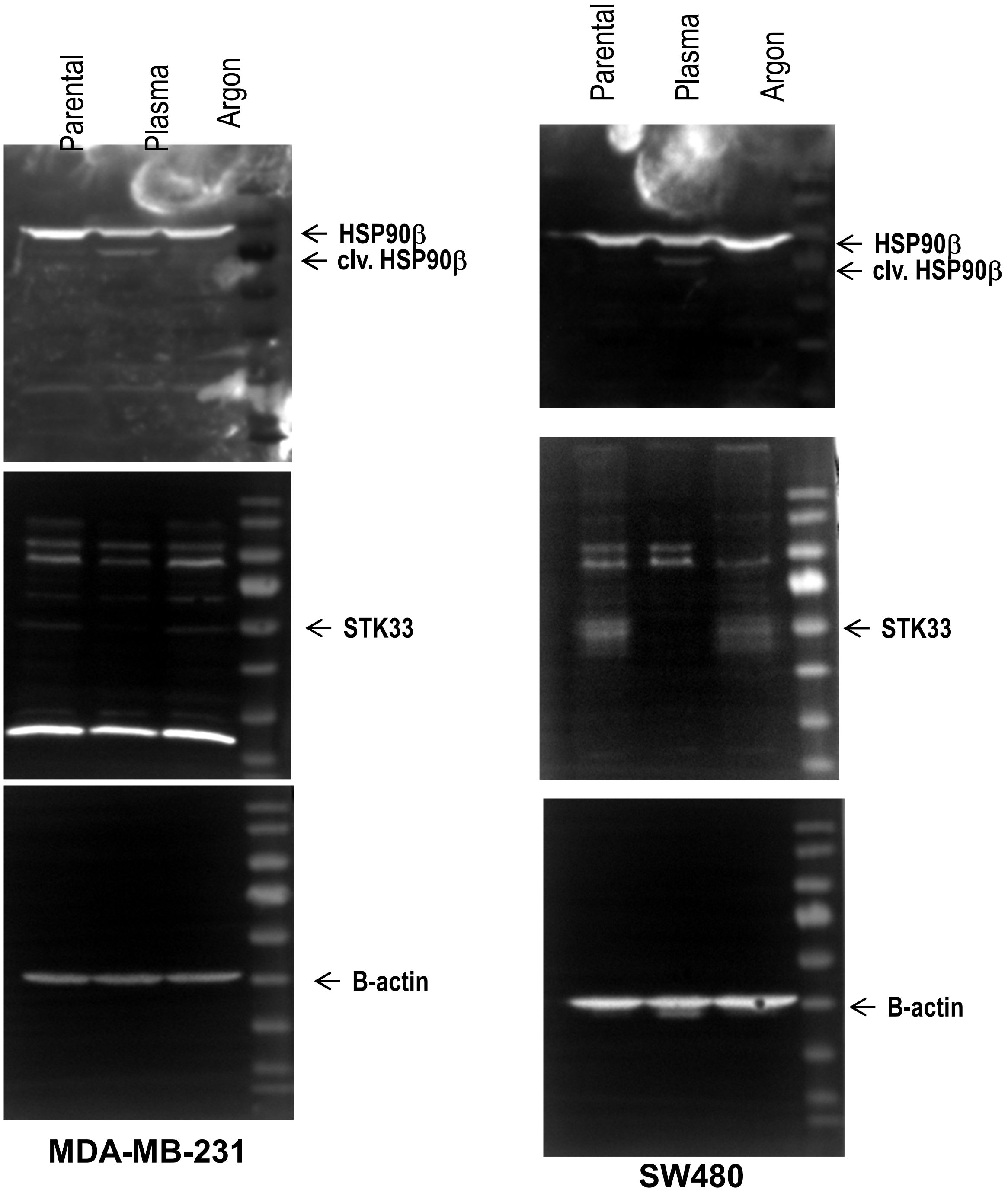

Suppl. Figure 3

Full blots to

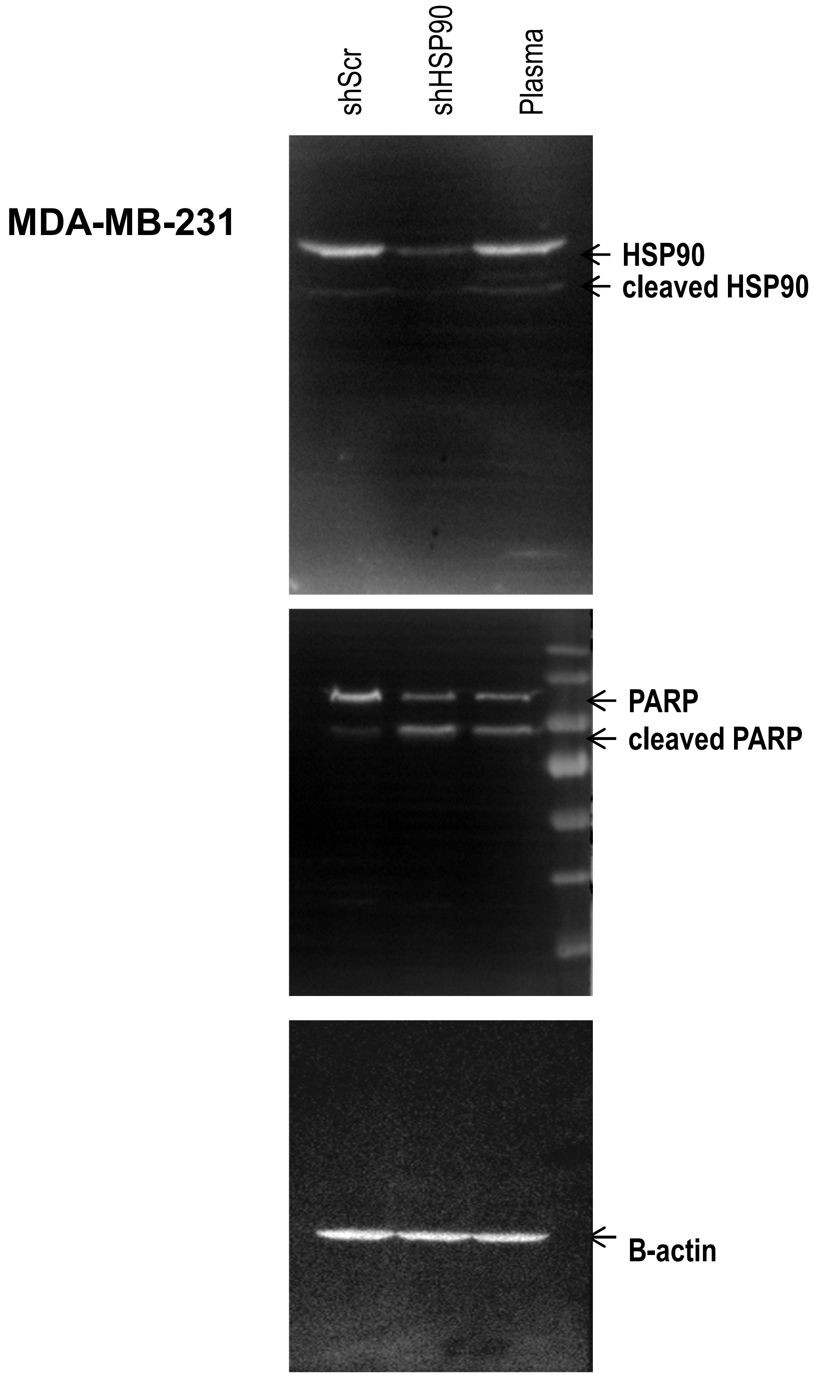

Suppl. Figure 4

Full blots to

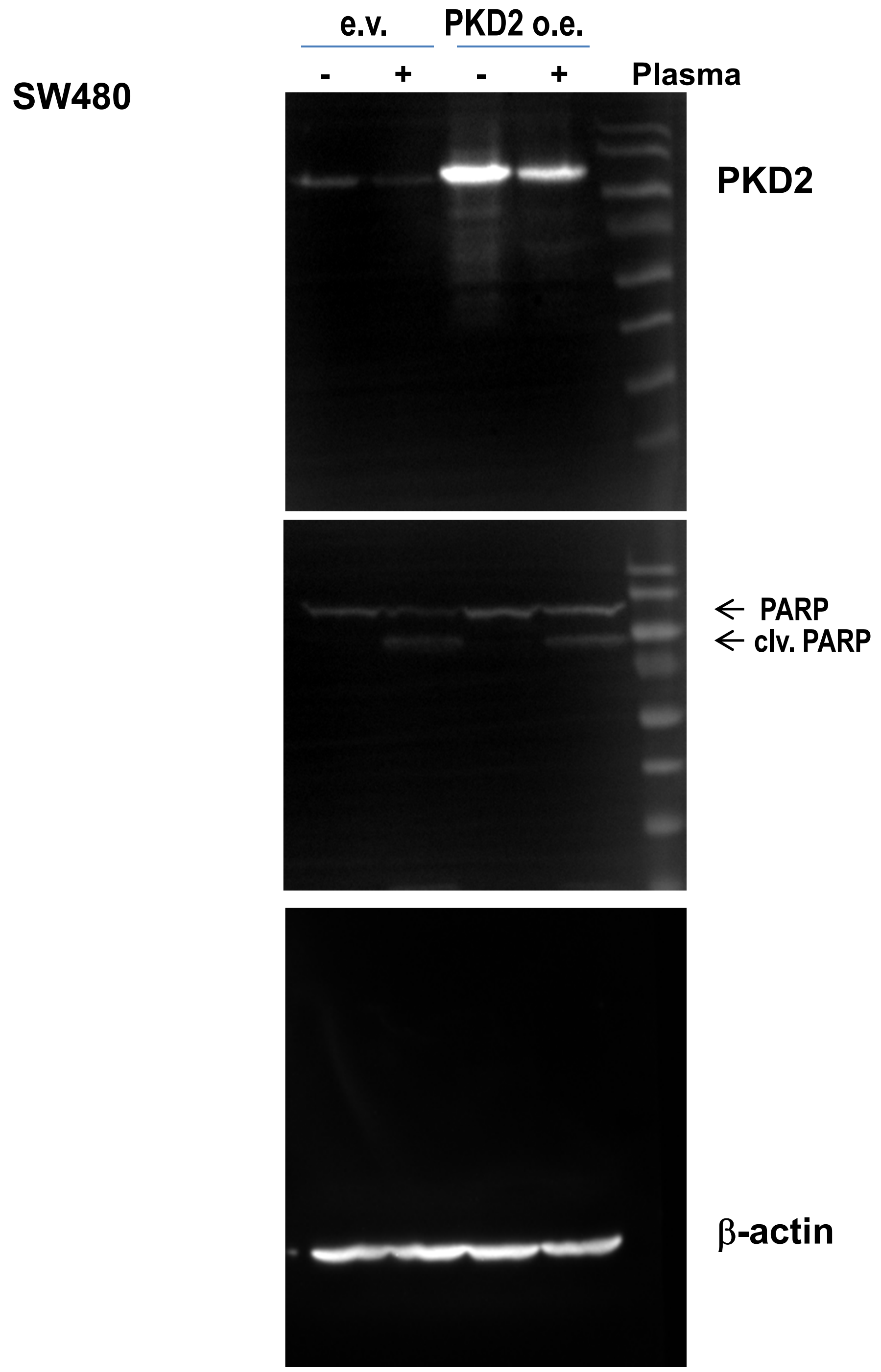

Supplement: Supplementary file 1 — Physical plasma-triggered ROS induces tumor cell death upon cleavage of HSP90 chaperone [file 41598_2019_38580_MOESM1_ESM.pdf]
